# Glass-Ceramics of the Lithium Aluminosilicate System Nucleated by TiO_2_: The Role of Redox Conditions of Glass Melting in Phase Transformations and Properties

**DOI:** 10.3390/ma18040785

**Published:** 2025-02-11

**Authors:** Stanislav Maltsev, Olga Dymshits, Irina Alekseeva, Anna Volokitina, Maksim Tenevich, Anastasia Bachina, Kirill Bogdanov, Svetlana Zapalova, Georgiy Shakhgildyan, Aleksandr Zhilin

**Affiliations:** 1 Institute of Machinery, Materials, and Transport, Peter the Great St. Petersburg Polytechnic University, St. Petersburg 195251, Russia; riplgandeleon@mail.ru; 2 S.I. Vavilov State Optical Institute, St. Petersburg 192171, Russia; vgolub@gmail.com (I.A.); anna.itmo@gmail.com (A.V.); zenii99@yandex.ru (S.Z.); 3 Ioffe Institute, St. Petersburg 194021, Russia; mtenevich@gmail.com (M.T.); a.k.bachina@yandex.ru (A.B.); 4 International Research and Education Center for Physics of Nanostructures, ITMO University, St. Petersburg 199034, Russia; kirw.bog@gmail.com; 5 Department of Glass and Glass-Ceramics, Mendeleev University of Chemical Technology, Moscow 125047, Russia; georgiy.shahgildyan@gmail.com; 6 D.V. Efremov Institute of Electrophysical Apparatus, St. Petersburg 189631, Russia; zhilin1311@yandex.ru

**Keywords:** redox conditions, titanium dioxide, nucleating agent, phase transformation, glass-ceramics, nanocrystals, Raman spectroscopy, absorption spectroscopy, coefficient of thermal expansion, X-ray diffraction analysis

## Abstract

Lithium aluminosilicate glasses nucleated by TiO_2_ are usually melted in oxidizing conditions. The reducing conditions of glass melting, which allow to obtain ions of variable valence in lower oxidation states, can influence the ability of titania to provide proper phase assemblage, structure and properties of lithium aluminosilicate glass-ceramics. The aim of this study is to reveal this influence. The model glass containing TiO_2_ was melted with and without the addition of As_2_O_3_. Using heat treatments between 680 °C and 1300 °C, XRD, SEM and DSC data, Raman and absorption spectroscopy, transparent glass-ceramics based on nanocrystals of β-quartz solid solutions (ss) and/or γ-Al_2_O_3_ with spinel structure and opaque glass-ceramics based on nanocrystals of β-spodumene ss were obtained and characterized. Three-phase immiscibility develops during secondary heat treatments. Al_2_TiO_5_ crystallizes from aluminotitanate amorphous regions simultaneously with the appearance of β-quartz ss, while traces of anatase and then rutile appear at elevated temperatures. Phase assemblage and the sequence of phase transformations do not depend on the redox conditions of glass melting, while the rate of these transformations is significantly higher in glass melted without the addition of As_2_O_3_. Absorption in glass melted without the addition of As_2_O_3_ and the corresponding glass-ceramics originate from octahedrally coordinated Ti^3+^ ions and Ti^3+^-Ti^4+^ pairs in glass and nanocrystals of γ-Al_2_O_3_, Al_2_TiO_5_ and β-quartz ss. Transparent glass-ceramics with a thermal expansion coefficient of ~0.3 × 10^−6^ K^−1^ were obtained from both glasses.

## 1. Introduction

Transparent lithium aluminosilicate glass-ceramics combine the unique properties of transparency and a close-to-zero thermal expansion coefficient [1,2,3]. Developed by Stookey more than 60 years ago [4], glass-ceramics of the lithium aluminosilicate system still remain a subject of intensive studies [5,6,7,8,9,10,11,12,13,14,15]. Stookey found out [4] that titania, TiO_2_, is an effective nucleating agent promoting crystallization of lithium aluminosilicate glasses of special compositions upon heat treatments. The role of titania as a nucleating agent of the lithium aluminosilicate system was studied by different structure-sensitive methods [16,17,18,19,20,21,22] and mechanisms controlling the formation of titania-doped glass-ceramics were suggested. It was demonstrated that they depend on the base glass composition, the nature and concentration of doping ions, and the amount of the nucleating agent. Being a polyvalent ion, titanium can be found in glasses in the form of Ti^4+^ and Ti^3+^ ions. Oxidation states of titanium ions depend on the glass melting temperature, oxygen partial pressure in the glass-melting furnace, the concentration of titanium ions and the glass composition [18,23,24,25,26,27,28,29]. Using titania as a nucleator, oxidizing agents are usually added to the glass batch to avoid unwanted coloration caused by traces of Ti^3+^ ions formed during the glass melting at high temperatures [1,2]. However, the reducing atmosphere of glass melting is required to obtain polyvalent ions in lower oxidation states. Therefore, it is worth knowing how the reducing atmosphere of glass melting influences phase transformations in glasses of the lithium aluminosilicate system nucleated by TiO_2_ and properties of corresponding glass-ceramics.

There are studies of phase transformations in titania-containing glasses of magnesium aluminosilicate [18,29,30], fresnoite [31,32,33], and zinc aluminosilicate [34] systems melted under different redox conditions. In these studies, it was demonstrated that variation of the glass melting conditions influences kinetics of liquid–liquid phase separation and of crystallization of metastable phases, compositions and structures of titania-containing phases and generally does not influence crystallization of the stable equilibrium phases [18,29,34]. The aim of the present work is to study the effect of redox conditions of glass melting on the phase composition and properties of lithium aluminosilicate glass-ceramics nucleated by titania, which will help to develop transparent thermal shock resistant glass-ceramics of the lithium aluminosilicate system containing polyvalent ions in lower oxidation states important for advanced photonic applications [35].

## 2. Materials and Methods

### 2.1. Materials Preparation

**Figure 1:**
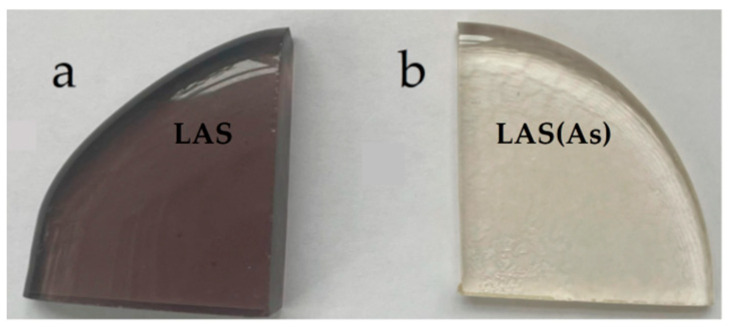
Photographs of as-casted glasses with thickness of ca. 6 mm: (**a**) LAS; (**b**) LAS_ox_. Glass notations are shown in figure.

Model glass with the composition 12 Li_2_O∙24 Al_2_O_3_∙64 SiO_2_ (mol%) [17,19] nucleated by 6 mol% TiO_2_ added on top of the base composition was melted in oxidizing (with addition of 0.5 wt% As_2_O_3_) and neutral (without addition of arsenic trioxide) conditions in a laboratory electric furnace in crucibles made of quartz ceramics at 1580 °C for 4 h (h) with stirring and casted onto a metal plate. The raw materials were reagent grade oxides and lithium carbonate manufactured by Neva-Reactive Co, Saint-Petersburg, Russian Federation. The weight of the glass batch was 400 g. The glasses were annealed at 640 °C for 1 h and cooled with an annealing furnace to room temperature. Small portions of the glasses were quenched by pressing with a metal plate and used for the differential scanning calorimetry (DSC) study. Annealed glasses were transparent and different in color. The glass melted under neutral conditions, referred to as LAS, exhibited a brownish-grey color, while the LAS_ox_ glass, prepared under oxidizing conditions, displayed a light-yellow tint. The color difference is clearly seen in photographs of the as-cast glasses with a thickness of ca. 6 cm (see Figure 1).

The glasses were cut into pieces with a diamond saw and isothermally heat-treated at temperatures between 680 °C and 1200 °C by single and two-stage heat treatments for 6 h at each stage.

### 2.2. Materials Characterization

#### 2.2.1. The Differential Scanning Calorimetry

The differential scanning calorimetry study was performed using a simultaneous thermal analyzer NETZSCH STA 449 F3 Jupiter, Netzsch Geratebau GmbH, Selb, Germany, with an Ar dynamic flow atmosphere. The temperature was increased from room temperature to 1300 °C at a heating rate of 10 °C⋅min^−1^. The quenched glasses and those subjected to single stage holdings at 680 °C, 700 °C, and 720 °C for 6 h, with a weight of about 15–20 mg were employed. To determine the character of crystalline phases responsible for exothermic effects on the DSC curves, quenched and heat-treated glasses with a weight of ca. 100 mg were heated in the furnace of the thermal analyzer up to corresponding temperatures. The glass-ceramics obtained were taken out from the cooled furnace and studied by X-ray diffraction (XRD) analysis.

#### 2.2.2. XRD Analysis

The XRD study of the powdered samples was performed using a Shimadzu XRD-6000 diffractometer, Shimadzu, Kyoto, Japan, with Cu Kα radiation and a Ni filter (λ = 1.5406 Å). The mean crystal sizes were calculated from the broadening of X-ray peaks according to Scherrer’s equation:

D = Kλ/Δ(2θ)cosθ,(1)

where λ is the wavelength of X-ray radiation, θ is the diffraction angle, Δ(2θ) is the width of peak at half of its maximum, and K is the constant assumed to be 1 [36]. The mean size of γ-Al_2_O_3_ crystals with a spinel structure was estimated using the peak with Miller’s indices *hkl* (440). The mean size of tieilite, Al_2_TiO_5_, crystals was determined from the peak with Miller’s indices (020). The mean size of crystals of β-quartz ss was determined from the peak with Miller’s indices (220). The mean size of crystals of β-spodumene ss was calculated using the peak with indices (102). An error of the calculation of the mean size of crystallites is about 5–10%.

The lattice parameter *a* of spinel nanocrystals was estimated from the position of the peak with *hkl* indices (440). The lattice parameters of Al_2_TiO_5_ were estimated using the peaks with indices (002), (020), (110), (023) and (200). The lattice parameters of β-quartz ss were calculated from the peaks with indices (110) and (211). The lattice parameters of β-spodumene ss were calculated from the peaks with *hkl* indices (111) and (102). An error in the estimation of the lattice parameters is ±0.003 Å.

#### 2.2.3. Raman Spectroscopy

Unpolarized Raman spectra were obtained using a backscattering configuration with a confocal InVia Renishaw Raman microscope, Renishaw, Wotton-under-Edge, England. The setup included a Leica objective with a magnification of ×50 (N.A. = 0.75), a thermoelectrically cooled CCD camera, and an edge filter. For excitation, a laser with a wavelength of 488 nm (from an Ar+ ion source) was employed. The spectral resolution achieved during the measurements was 2 cm^−1^. Each spectrum was obtained by averaging data from 10 acquisitions, each lasting 10 s. The samples were either transparent polished flat-parallel plates having a thickness of ~1 mm or opaque pieces of materials.

#### 2.2.4. Scanning Electron Microscopy

The morphology of the initial and heat-treated glasses was obtained by scanning electron microscopy (SEM) using a Tescan Vega 3 SBH microscope, Tescan, Brno, Czech Republic. For the study, the surfaces of the same transparent polished plates and opaque bulk samples, which were used for measuring Raman spectra, were preliminarily cleaned in isopropyl alcohol and benzene, etched in a solution of HF for about 2 s and washed in distilled water. ImageJ software version 1.51j8 [37] was used to calculate the particle sizes.

#### 2.2.5. Absorption Spectroscopy

A Shimadzu UV-3600 spectrophotometer, Shimadzu, Japan, was employed for recording absorption spectra within the wavelength range from 250 to 3300 nm using the same polished ~1 mm thick flat-parallel plates.

#### 2.2.6. The Linear Coefficient of Thermal Expansion

The thermal expansion coefficients of the glasses and glass-ceramics were estimated using a Linseis L 75 vs. 1000 dilatometer, Linseis Messgeräte GmbH, Selb, Germany. The samples were rods with a length ranging from 30 to 50 mm and flat-parallel planes with sections measuring ~5 × 5 mm. The measurements were performed at room temperature up to 320 °C with a heating rate of 5 °C⋅min^−1^.

## 3. Results

**Figure 2:**
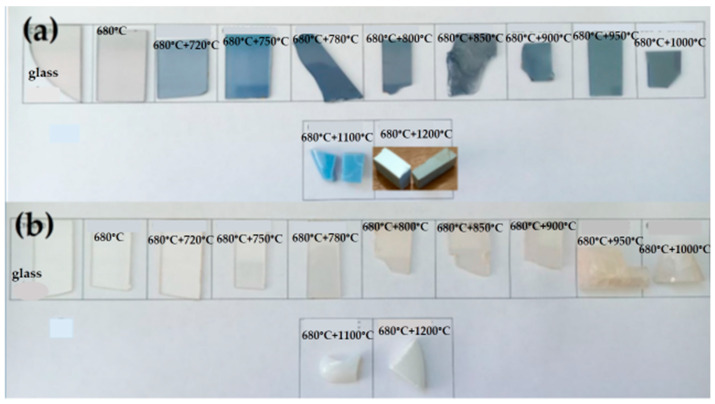
Photographs of samples of the precursor glasses, glasses heat-treated at 680 °C and glass-ceramics prepared by heat treatments with the first stage at 680 °C and the second stage between 720 °C and 1200 °C: (**a**) LAS; (**b**) LAS_ox_. The regimes of heat treatments are shown in figure. Heat-treatment duration at each stage is 6 h. The thickness of transparent polished plates is 1 mm.

The polished plates of transparent glasses and glass-ceramics and the pieces of opaque glass-ceramics are shown in Figure 2. Note that the thin polished plate of the glass made from the brownish-grey as-cast LAS glass has a light grey coloration because of a six times smaller thickness. The initial glasses, heat-treated at 680 °C for 6 h and by two-stage heat treatments with the second hold at temperatures between 720 °C and 900 °C, are transparent. The LAS glass-ceramics obtained at 950 °C and at 1000 °C are also transparent, while LAS_ox_ glass-ceramics obtained at these temperatures are translucent. Both glass-ceramics obtained at 1100 °C are translucent, and those prepared at 1200 °C are opaque. Therefore, glasses melted under neutral conditions lose transparency at higher secondary heat treatment temperatures than glasses melted under oxidizing conditions. The opaque LAS glass-ceramic prepared at 1200 °C is grey colored and has a white surface layer, see Figure 2a.

### 3.1. Study by Differential Scanning Calorimetry

**Figure 3:**
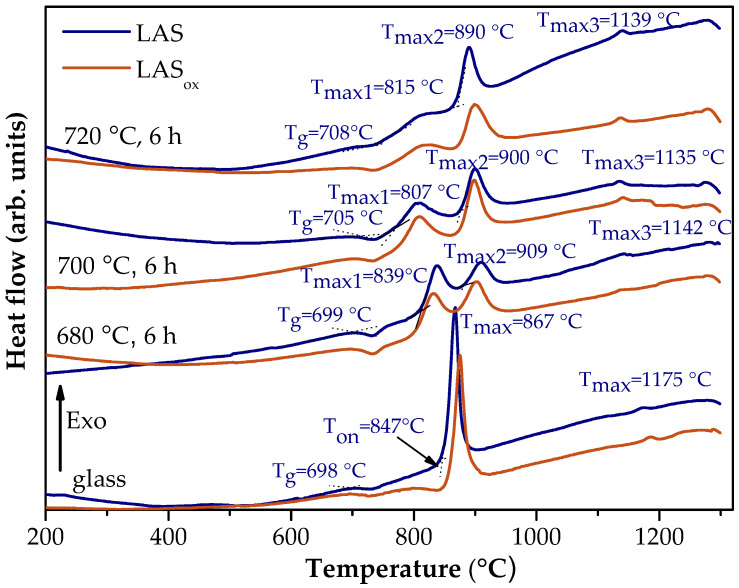
DSC signals as time functions for quenched and heat-treated LAS and LAS_ox_ glasses. T_g_ denotes the glass transition temperature, T_on_ is the onset crystallization temperature, T_max_ is the maximum crystallization temperature. The curves are shifted for the convenience of observation.

Figure 3 shows the DSC scans of the quenched LAS and LAS_ox_ glasses, as well as these glasses heat-treated at 680 °C, 700 °C and 720 °C for 6 h. The shapes of the corresponding DCS curves of LAS and LAS_ox_ glasses are nearly similar, being different in the values of characteristic crystallization temperatures, see Figure 3 and Table 1. The DSC curve of the quenched LAS_ox_ glass shows three peaks with markedly different intensities. The first one is a broad peak of a low intensity with T_max_ = 802 °C, the second one is a narrow and intense peak with crystallization onset temperature T_on_ = 851 °C, and crystallization maximum temperature T_max_ = 874 °C. The third one is a weak exothermic peak with T_max_ = 1186 °C. The first peak is usually assigned to nucleation of the main crystalline phase, the second one to the crystallization of the lithium aluminosilicate solid solution (ss) with β-quarts structure (β-quarts ss), and the third one to the transformation of β-quartz ss into β-spodumene ss [38,39]. The DSC curve of the LAS glass exhibits a plateau instead of the first peak, see Figure 3 and Table 1. All characteristic crystallization temperatures of the LAS glass are lower than those for the LAS_ox_ glass, while the glass transition temperatures of both glasses are similar, see Table 1. Therefore, the redox conditions of glass melting affect crystallization temperatures and do not affect the transition temperature of glasses.

Preliminary heat treatments lead to a drastic change in the appearance of DSC curves, see Figure 3. The character of this change for LAS and LAS_ox_ samples is the same, differing in the temperatures of thermal effects. Instead of a single narrow intense peak, two broad exothermic peaks of lower intensities appear. The characteristic temperatures for both glasses preliminarily heat-treated at 680 °C, 700 °C, and 720 °C are shown in Figure 3 and Table 1. T_max_ of the second broad exothermic peak is 35–40 °C higher than this temperature for the initial quenched glass. With the increase in the temperature of preliminary heat treatment, there is a redistribution of intensities of these two peaks in favor of the second one, see Figure 3. T_max_ of the second peak and T_g_ gradually increase, see Table 1.

The reason for this behavior will be explained below while discussing the phase composition of the samples. The characteristic T_g_, T_on_ and T_max_ temperatures of glasses preliminary heat-treated at 680 °C are similar, being somewhat higher for the LAS glass than for the corresponding LAS_ox_ samples.

**Figure 4:**
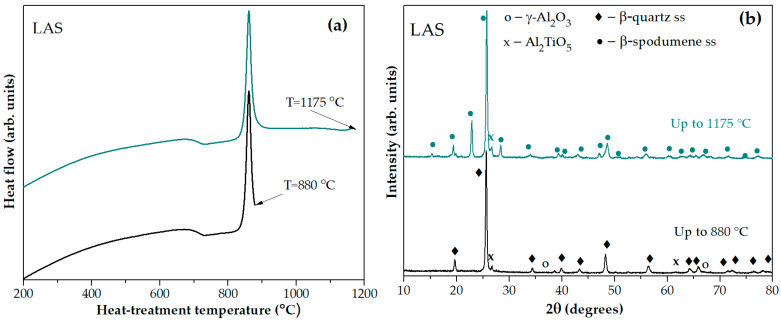
(**a**) DSC signals as time functions for the quenched LAS glass with stops at temperatures of 880 °C and 1175 °C; (**b**) XRD patterns of thus obtained LAS glass-ceramics. The curves are shifted for the convenience of observation.

The XRD pattern of the glass-ceramic obtained by heating the quenched LAS glass in the furnace of the DSC instrument up to the temperature of the sharp exothermic peak at 880 °C, see Figure 4a, testifies to the crystallization of β-quarts ss, traces of tieilite, Al_2_TiO_5_, and spinel, see Figure 4b. On the XRD pattern of the sample heated up to the temperature of the third exothermic effect at 1175 °C, additional diffraction peaks appear as compared with the pattern of the sample heated up to 880 °C, manifesting in a transformation of the β-quarts ss into the β-spodumene ss while spinel traces disappeared. Thus, the small high-temperature peak evolving at ~1175 °C is due to crystallization of β-spodumene ss, see Figure 4b.

**Figure 5:**
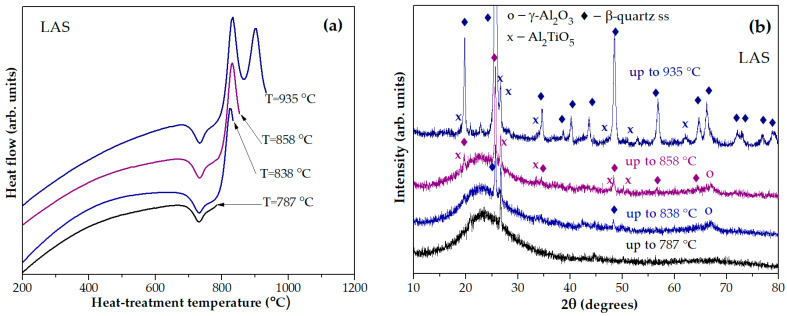
(**a**) DSC signals as time functions for the LAS glass preliminary heated at 680 °C for 6 h with stops at temperatures of 787 °C, 838 °C, 858 °C, and 935 °C; (**b**) XRD patterns of thus obtained LAS glass-ceramics. The curves are shifted for the convenience of observation.

Figure 5a shows the DSC signals of the LAS glass preliminarily heat-treated at 680 °C for 6 h with stops at temperatures of 787 °C, 838 °C, 858 °C, and 935 °C. The corresponding XRD patterns are presented in Figure 5b. The material obtained by the heat treatment at 680 °C, 6 h + 787 °C, 0 h is X-ray amorphous. The sample obtained by the heat treatment at 680 °C, 6 h + 838 °C, 0 h contains nanocrystals of γ-Al_2_O_3_ with a spinel structure and traces of β-quarts ss. It is speculated that this exothermic peak is caused by the crystallization of spinel, while traces of β-quarts ss appear during the cooling of the sample in the furnace, because the samples heated to the specified temperature could not be immediately removed from the hot furnace of the DSC instrument. The XRD pattern of the sample obtained by the heat treatment at 680 °C, 6 h + 858 °C, 0 h, corresponding to the end of the first exothermic peak and the beginning of the second one, contains peaks of nanocrystals of γ-Al_2_O_3_, Al_2_TiO_5_ and β-quarts ss. The XRD pattern of glass-ceramic obtained by the heat treatment at 680 °C, 6 h + 935 °C, 0 h proves the crystallization of β-quarts ss and Al_2_TiO_5_. The presence of γ-Al_2_O_3_ cannot be ruled out on this XRD pattern because the most intense peak of γ-Al_2_O_3_ overlaps the peaks of β-quarts ss and is therefore difficult to detect.

**Figure 6:**
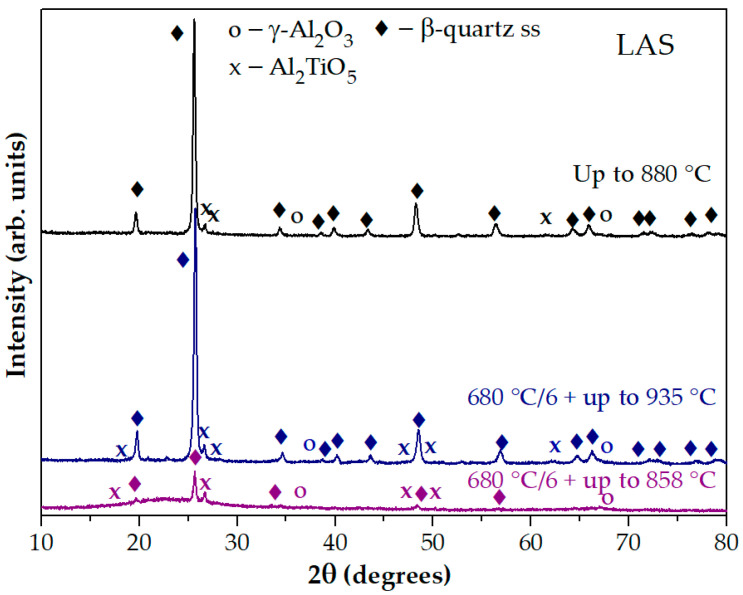
XRD patterns of the LAS quenched glass, and the glass heat-treated at 680 °C for 6 h and then heated in the furnace of the DSC instrument. The heat-treatment schedules are shown in the figure. The patterns are shifted for the convenience of observation.

The XRD pattern of the glass preliminarily heat-treated at 680 °C for 6 h and heated in the DSC furnace up to 858 °C is significantly different from the XRD pattern of the quenched glass heated in the DSC furnace up to 880 °C. The XRD pattern of the glass preliminarily heat-treated at 680 °C for 6 h and heated in the DSC furnace up to 935 °C is similar to the XRD pattern of the quenched glass heated in the DSC furnace up to 880 °C, see Figure 6. Thus, the first peak on the DSC curve of the preliminary heat-treated sample is associated with the crystallization of the phase with the spinel structure, and the second peak is caused by the crystallization of β-quartz ss and Al_2_TiO_5_. On the DSC curve of the quenched sample there is no exothermic peak associated with the crystallization of the phase with spinel structure. Therefore, preliminary heat treatments provoke spinel crystallization and increase the crystallization temperature of β-quartz ss and Al_2_TiO_5_.

### 3.2. XRD Study

**Figure 7:**
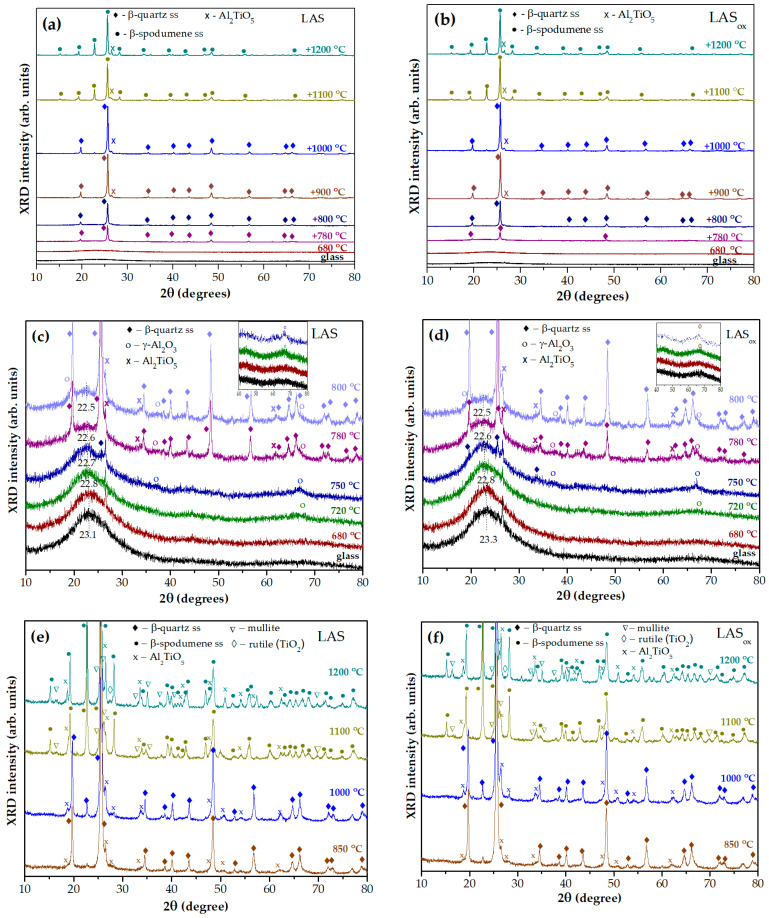
(**a**,**b**) XRD patterns of LAS and LAS_ox_ glasses and glass-ceramics: (**a**) the LAS; (**b**) the LAS_ox_; (**c**,**d**) a closer look on XRD patterns of glasses and glass-ceramics obtained by heat treatments at the second stage up to 800 °C: (**c**) the LAS; (**d**) the LAS_ox_; (**e**,**f**) a closer look on XRD patterns of glass-ceramics obtained by heat treatments at the second stage between 850 °C and 1200 °C: (**e**) the LAS; (**f**) the LAS_ox_. The heat-treatment temperature at the first stage is 680 °C, duration of each stage is 6 h. The patterns are shifted for the convenience of observation.

Figure 7a,b shows XRD patterns of the LAS and LAS_ox_ glasses and glasses of the same compositions, which underwent heat treatment at 680 °C and two-stage heat treatments with a temperature at the second stage between 720 °C and 1200 °C. The holding time at each stage was 6 h. The figures allow the following of the formation of the main crystalline phase, β-quartz ss, at temperatures between 780 °C and 1000 °C, and crystallization of β-spodumene ss at temperatures between 1100 °C and 1200 °C. Figure 7c,d gives a closer look at the XRD patterns of the glasses and glass-ceramics fabricated by heat treatments at the second stage up to 800 °C. Initial LAS and LAS_ox_ glasses are X-ray amorphous with a maximum amorphous halo located at 2θ = 23.1° and 23.3°, respectively. Glasses heat-treated at the nucleation stage of 680 °C for 6 h are X-ray amorphous, and their XRD patterns are similar to those of glass precursors. The two-stage heat treatment with the second stage at 720 °C for 6 h leads to crystallization of a small fraction of crystallites with spinel structure, manifested by an appearance of a broad peak with Miller indices *hkl* (440) at 2θ ≅ 66.8°.

Its low intensity prevents estimation of the lattice parameter and the size of the spinel crystallites. The position of the amorphous halo shifts to a smaller angle 2θ = 22.8° for both samples. After the second stage heat treatment at 750 °C for 6 h, spinel peaks become more pronounced, which indicates an increase in the volume fraction of this phase. The lattice parameters of spinel nanocrystals are a_norm_ = 7.918 Å and a_ox_ = 7.916 Å, and sizes are D_norm_ = 4.5 nm and D_ox_ = 7.3 nm, respectively. Traces of β-quartz ss are also seen on the patterns. The appearance of spinel and traces of β-quartz ss cause a further change in the composition of the residual glass phase, which is manifested by a shift in the maximum of the amorphous halo to smaller angles, indicating that the residual glass becomes enriched in silica. After heat treatments at the second stage at 780 °C and 800 °C, the β-quartz ss becomes the predominant crystalline phase, while spinel and tieilite, Al_2_TiO_5,_ nanocrystals are also found in the XRD patterns. The position of the amorphous halo of residual glass is shifted to 2θ = 22.6°and then to 2θ = 22.5° with increasing heat-treatment temperature, see Figure 7c,d.

The lattice parameters and mean crystallite sizes of spinel obtained at different heat-treatment temperatures are presented in Table 2. It is believed that spinel has the composition and structure of γ-Al_2_O_3_. The lattice parameter *a* of the unit cell of the cubic modification of Al_2_O_3_ usually takes the value *a* = 7.900–7.908 Å [40]. With a slight oxygen deficiency, the parameter *a* of γ-Al_2_O_3_ becomes equal to 7.911 Å (ICDD PDF card #79-1558) and 7.914 Å (ICDD PDF card 79-1557) [41]. The lattice parameter *a* of γ-Al_2_O_3_ crystallites in glass-ceramics changes from 7.915 Å to 7.925 Å and increases with increasing the heat-treatment temperature. The difference in the parameter *a* of γ-Al_2_O_3_ nanocrystals in LAS and LAS_ox_ glass-ceramics is very small. Nevertheless, the lattice parameter *a* of γ-Al_2_O_3_ in LAS glass-ceramics is slightly higher than in LAS_ox_ ones. The sizes of spinel crystallites increase with increasing the heat-treatment temperature ranging from 4.5 to 14.0 nm, see Table 2.

**Figure 8:**
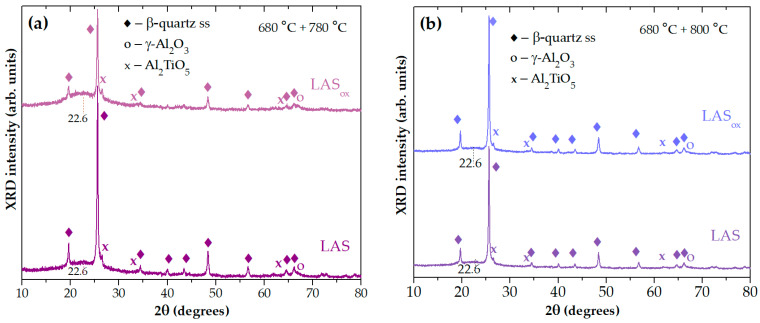
XRD patterns of the LAS and the LAS_ox_ glass-ceramics obtained by two-stage heat treatments at: (**a**) 680 °C, 6 h + 780° C, 6 h; (**b**) 680 °C, 6 h + 800 °C, 6 h. The patterns are shifted for the convenience of observation.

Figure 8a shows that in spite of the same phase assemblage of glass-ceramics obtained by two-stage heat treatment at 680 °C, 6 h + 780 °C, 6 h, the crystallinity fractions in the LAS glass-ceramic is much higher than in the LAS_ox_ one. This difference is levelled out by the heat treatment at 680 °C, 6 h + 800 °C, 6 h, see Figure 8b.

Figure 7e,f show the XRD patterns of glass-ceramics obtained by two-stage heat treatments with a second-stage temperature between 850 °C and 1200 °C. After the heat treatment at 850 °C the crystallinity fraction increases to such extent that the amorphous halo disappears. Volume fractions of β-quartz ss and Al_2_TiO_5_ gradually increase. β-spodumene ss crystallizes at the expense of β-quartz ss at 1100 °C. Mullite (ICDD PDF card # 79-1458) appears at the same heat-treatment temperature. The lattice parameters and mean crystallite sizes of β-quartz ss and β-spodumene ss are presented in Table 2. Their variation with heat-treatment temperature is similar for the LAS and the LAS_ox_ glasses.

As was mentioned above, the appearance of the surface and the volume of the LAS glass-ceramics obtained by the heat treatment at 1200 °C are different. The surface is a dense, white-colored layer while the volume is grey colored, see Figure 2. Traces of rutile (ICDD PDF card #78-1509) are found on the XRD patterns taken from the grey part of the LAS glass-ceramic. The appearance of rutile is accompanied by some decrease in the tieilite fraction. We failed to find a difference in the phase assemblages of the surface and volume of LAS_ox_ glass-ceramic obtained by the same heat treatment. The XRD pattern shows a tiny fraction of rutile in this sample.

Let us discuss the features of crystallization of tieilite, Al_2_TiO_5_. As mentioned above, the onset of the crystallization of β-quartz solid solution in glasses at a temperature of 780 °C is accompanied by the appearance of a small fraction of Al_2_TiO_5_ crystallites with mean sizes of ~6 and 8 nm for LAS and LAS_ox_ glass-ceramics, respectively, see Table 3. Increasing the heat-treatment temperature up to 1200 °C leads to an increase in the size of Al_2_TiO_5_ crystallites up to ~40 nm, a slow increase in their fraction and a change in their lattice parameters, see Table 3. The values of the lattice parameter do not change with the heat-treatment temperature within the measurement error and are independent of the redox conditions of glass melting. Lattice parameters b, c and the volume increase with the heat-treatment temperature. An analysis of the evolution of lattice parameters shows that the lattice of a Al_2_TiO_5_ unit cell, which has the shape of a rectangular parallelepiped elongated in [010] and [001] directions, becomes even more elongated in the same directions under the influence of high temperature. The lattice parameters of tieilite in LAS and LAS_ox_ glass-ceramics are similar. The character of their variation with temperature is also similar. The redox conditions of glass melting do not significantly affect the structural transformations in tieilite crystallites. A pronounced change in lattice parameters after the heat treatment at 1200 °C found in the present study can be a prerequisite to decomposition of tieilite with exsolution of rutile [42]. Indeed, as mentioned above, traces of rutile are found in LAS_ox_ and in the volume of the LAS glass-ceramics obtained by the heat treatment at 1200 °C. The comparison of XRD patterns of the LAS and the LAS_ox_ glass-ceramics obtained by the heat treatment at 1200 °C demonstrates that LAS_ox_ glass-ceramic contains a smaller fraction of rutile and a higher fraction of mulite than the LAS glass-ceramic, compare Figure 7e,f.

The sequence of phase transformations revealed by XRD study is similar for both glasses: precursor glasses and glasses subjected to the nucleation treatment are X-ray amorphous; nanocrystals of γ-Al_2_O_3_ with spinel structure and sizes ranging from 4.5 nm to 14 nm evolve during heat treatments in the second stage in the temperature range from 720 °C to 800 °C; β-quartz ss and the crystalline phase of the nucleating agent, tieilite, appear additionally to spinel during the heat treatment at 780 °C; β-quartz ss are obtained by heating in the interval between 780 °C and 1000 °C; the glass-ceramic prepared at 1100 °C contains β-spodumene ss instead of β-quartz ss and traces of mullite; β-spodumene ss is the main crystalline phase of glass-ceramics produced in the temperature range between 1100 °C and 1200 °C; tieilite nanocrystals with a mean size ranging from ~6 nm to ~45 nm are found in glass-ceramics prepared in the temperature interval from 800 °C to 1200 °C; during the heat treatment at 1200 °C, the rutile crystallites appear in the phase assemblage of glass-ceramics.

In spite of the same phase assemblage of glass-ceramics prepared from glasses melted in different redox conditions, the kinetics of phase transformations and spinel lattice parameters are slightly different. The spinel lattice parameters in the LAS glass-ceramics are larger than in the LAS_ox_ glass-ceramics. The crystallinity fractions in the LAS glass-ceramic obtained by two-stage heat treatment at 680 °C, 6 h + 780 °C, 6 h is much higher than in the LAS_ox_ one. However, this difference is levelled out by the heat treatment at 680 °C, 6 h + 800° C, 6 h. The rutile crystallinity fraction in the LAS glass-ceramics is larger than in the LAS_ox_ one, while the mullite fraction is smaller.

### 3.3. Study by Raman Spectroscopy

**Figure 9:**
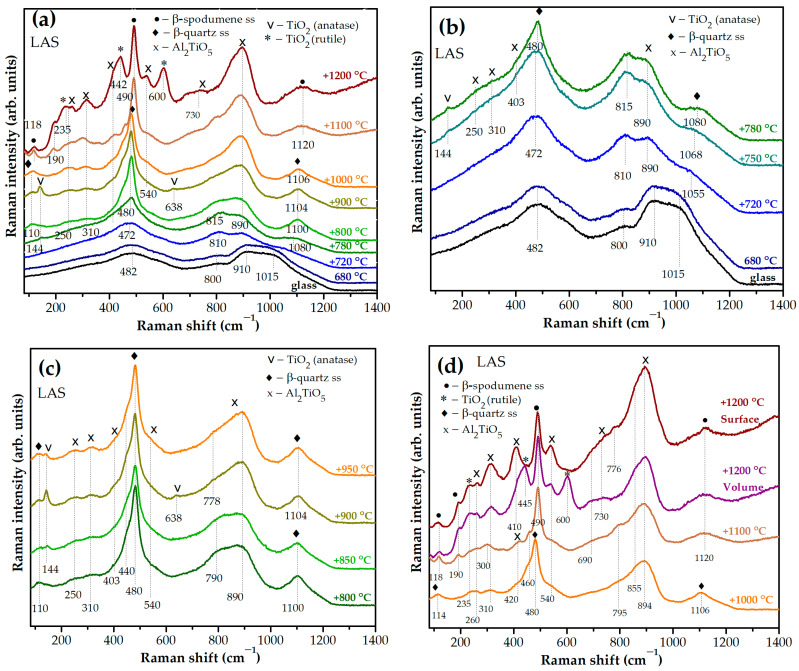
Raman spectra of the LAS glass and glass-ceramics obtained by single and two-stage heat treatments: (**a**) from the glass to 1200 °C; (**b**) from the glass up to 780 °C; (**c**) from 800 °C up to 950 °C; (**d**) from 1000 °C up to 1200 °C. The time of heat treatment at each hold is 6 h. The spectra are shifted for the convenience of observation.

Figure 9a,b show Raman spectra of the initial and heat-treated LAS glass. Raman spectra of initial LAS and LAS_ox_ glasses are similar and contain broad bands with maxima at 482 cm^−1^, 800 cm^−1^, 910 cm^−1^ and ~1015 cm^−1^. The wing of the latter band extends to 1200 cm^−1^. A similar spectrum was obtained in ref. [19] for the glass of the same composition nucleated by 7 mol% TiO_2_ and melted in oxidizing conditions. The bands at 482 cm^−1^, 800 cm^−1^, and ~1000–1200 cm^−1^ are due to vibrations of bonds in the tetrahedrons of the aluminosilicate network in the glass structure [43], and the band at ~910 cm^−1^ is due to vibrations of [TiO_4_] tetrahedra embedded in this network [44]. After the heat treatment at the nucleation stage at a temperature of 680 °C, minor changes are noticed in the Raman spectrum, see Figure 9a,b. The high-frequency band slightly broadens and the band with a maximum at 800 cm^−1^ somewhat increases in intensity compared to the band with a maximum at 910 cm^−1^, which indicates the development of liquid–liquid phase separation of the precursor glass [19]. The position of the band at 482 cm^−1^ is not influenced by this heat treatment.

After the two-stage heat treatment with a temperature of 720 °C at the second stage, significant changes are observed in the Raman spectrum, see Figure 9a,b. The bands with maxima at 910 cm^−1^, 800 cm^−1^, and 482 cm^−1^ move their positions to 890 cm^−1^, 810 cm^−1^, and 472 cm^−1^, respectively. Instead of the band with a maximum at ca. 1015 cm^−1^, a band at 1055 cm^−1^ appears. The intensity of the band at 472 cm^−1^ increases as compared with the similar band in spectra of precursor glass and glass heat treated at the nucleation stage. The intensity of the band at ~810 cm^−1^ increases relatively to the band at ca. 890 cm^−1^. The increase in the intensity of this band at the expense of the band with a maximum at ca. 890 cm^−1^ is due to the superposition of vibrations of the [TiO_5_] and [TiO_6_] groups in amorphous aluminotitanate regions on a weak band in the 800 cm^−1^ region, corresponding to vibrations of the tetrahedrons of the aluminosilicate network [17,19,20]. These changes are associated with a further development of the liquid–liquid phase separation with the formation of aluminotitanate amorphous regions [19,20] and a change in the composition of the aluminosilicate glass network, which is in accordance with a change in the position of the amorphous halo in the corresponding XRD pattern, see Figure 7c. Crystallization of γ-Al_2_O_3_ with spinel structure revealed by XRD analysis does not show itself in the Raman spectrum of this glass-ceramic. Running ahead, one will say that spectral features of γ-Al_2_O_3_, were not detected in Raman spectra of samples obtained by heat treatments between 750 °C and 800 °C as well, in spite of the fact that XRD analysis reliably identified them. The reason is that bond vibrations in aluminate spinel crystals are very weak compared to bond vibrations in titanium-containing compounds. According to refs [45,46], γ-Al_2_O_3_ exhibits narrow peaks with maxima at 315, 410, 520, 713 and 835 cm^−1^ in the Raman spectrum of γ-Al_2_O_3_ corresponding to vibrations of the Al–O bond in tetrahedral structural units of AlO_4_ [45]. Based on calculations presented in ref. [47], the strongest Raman peak for γ-Al_2_O_3_ with a spinel structure is located at ~401 cm^−1^. There are also several bands of medium intensities ranging from 100 cm^−1^ to 900 cm^−1^ [47]. In Raman spectra presented in Figure 9b there is a band with a maximum at 403 cm^−1^, which could be the spectroscopic sign of γ-Al_2_O_3_. However, this band can be also caused by vibrations in tieilite crystals, because this band appears simultaneously with other bands at 250 cm^−1^, 310 cm^−1^, and 890 cm^−1^, characteristic of tieilite, which crystallizes at a larger temperature interval of heat treatments than γ-Al_2_O_3_ (see below). It is worth mentioning that the spectroscopic signs of crystals with a spinel structure were found only in Raman spectra of spinel-based glass-ceramics of magnesium [48] and zinc aluminosilicate systems [34], where the spinel crystallinity fraction was significantly higher than in the present case.

In the Raman spectrum of the glass-ceramic obtained by the heat treatment at the second stage at 750 °C, the bands with maxima at 472 cm^−1^ and 815 cm^−1^ are enhanced, positions of the band maxima change from 810 cm^−1^ to 815 cm^−1^ and from 1055 cm^−1^ to 1068 cm^−1^, and a very weak band appears at ~144 cm^−1^. The latter peak can be attributed to the most intense vibration in the Raman spectrum of the metastable modification of TiO_2_, anatase [17].

In the Raman spectrum of the sample obtained by the heat treatment at the second stage at a temperature of 780 °C, the band at ~144 cm^−1^, caused by vibrations in anatase nanocrystals, narrows, the band at 480 cm^−1^ narrows and intensifies, and the band at ~1080 cm^−1^ appears. The last two bands are related to vibrations in nanocrystals of β-quartz ss [17,19,49,50,51]. As mentioned above, in the spectrum of these glass-ceramics there is a number of bands with maxima at 250 cm^−1^, 310 cm^−1^, 403 cm^−1^, 540 cm^−1^, and 890 cm^−1^. They appear simultaneously, and their intensities increase continuously with the increasing temperature of heat treatment. These bands belong to vibrations in tieilite nanocrystals [17,19], which is in accordance with the XRD data. The Raman spectrum of glass-ceramic obtained by heat treatment with a temperature of 900 °C at the second stage shows two anatase bands at ~144 cm^−1^ and ~638 cm^−1^ manifesting the maximum anatase crystallinity fraction achieved by this heat treatment, see Figure 9a,c. After increasing the temperature of the second hold to 1000 °C, the bands related to anatase disappear. Note that the XRD peaks of anatase coincide in position with the main peaks of β-quartz ss and its small amount cannot be detected by the XRD data [17,19].

As the temperature at the second stage of heat treatment increases, the intensities of the bands at ~460 cm^−1^ and ~1080 cm^−1^ increase, and their peaks become narrower, see Figure 9c, which indicates the development of β-quartz ss crystallization. Their positions constantly move to longer wave numbers manifesting enrichment of these crystals with silica [50]. After the second stage heat treatment at 1100 °C, these bands shift to 490 cm^−1^ and 1120 cm^−1^, which can be interpreted as recrystallization of β-quartz ss into β-spodumene ss [17,19]. Starting from the heat treatment at 800 °C, a weak band appears at 110 cm^−1^ simultaneously with increasing intensity and narrowing of the bands attributed to β-quartz ss. This band can correspond to external vibrations in nanocrystals of β-quartz ss [51]. In Raman spectra of glass-ceramics obtained by heat treatments at 1100 °C and at 1200 °C, weak bands at ~118 cm^−1^ and ~190 cm^−1^ were observed that can be attributed to external vibrations in nanocrystals of β-spodumene ss [49], see Figure 9d.

**Figure 10:**
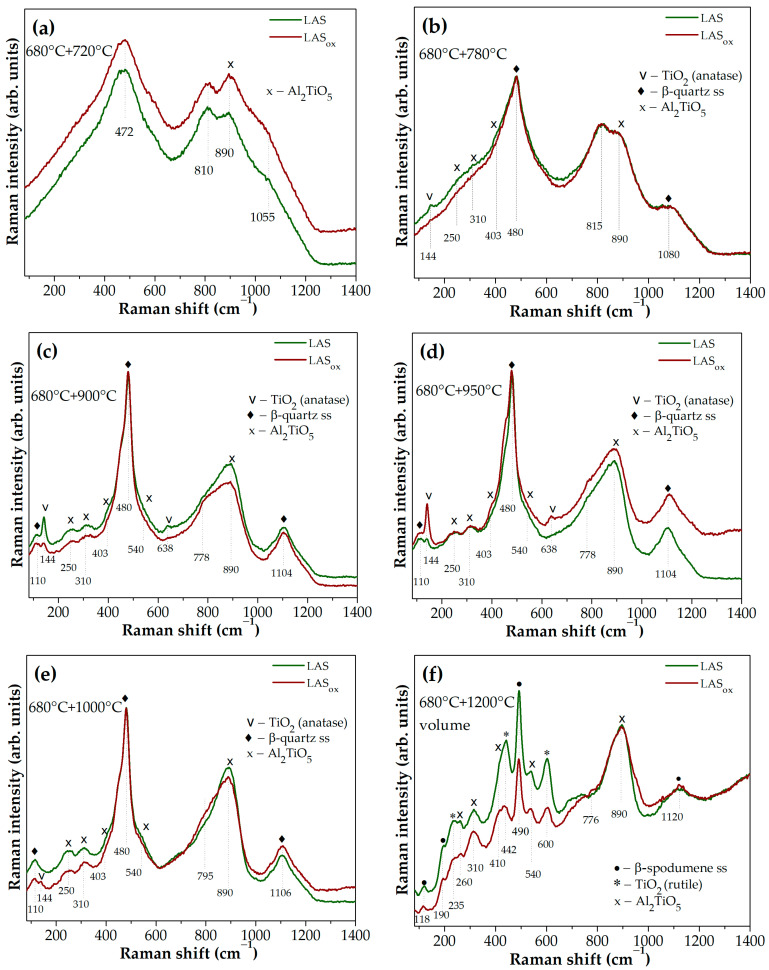
Raman spectra the LAS and the LAS_ox_ glass-ceramics obtained by two-stage heat treatments at: (**a**) 680 °C + 720 °C; (**b**) 680 °C + 780 °C; (**c**) 680 °C + 900 °C; (**d**) 680 °C + 950 °C; (**e**) 680 °C + 1000 °C; (**f**) 680 °C + 1200 °C. The timeof heat treatment at each stage is 6 h. The spectra are shifted for the convenience of observation.

Figure 9d shows the bands at ~260 cm^−1^, ~445 cm^−1^ and ~600 cm^−1^, which are related to crystallization of rutile [17] at the expense of tieilite in the bulk of glass-ceramic obtained by the heat treatment at a second stage at 1200 °C. The signs of rutile crystals were not found in the spectrum from the white surface of this sample, which is in accordance with XRD data. XRD patterns of glass-ceramics obtained by heat treatments at 1100 °C and 1200 °C contain small mullite fractions, see Figure 7e,f. Raman spectra of synthetic mullites of different compositions have similar spectra with the strongest bands located at 415 cm^−1^, 600 cm^−1^, and 980 cm^−1^ [52]. They can be hidden in the contours of the corresponding broad bands in Raman spectra, see Figure 10f.

Raman spectra of glass-ceramics obtained by the same heat treatments that differ most strongly from each other according to Raman spectroscopy data are presented in Figure 10a–f. Raman spectra of glass precursors, as well as the spectra of glasses heat-treated at 680 °C for 6 h are similar to each other. The spectra of glass-ceramics obtained by the two-stage heat treatment at 680 °C and at 720 °C for 6 h differ from each other by the ratio of band intensities in the region of high wave numbers, see Figure 10a. The increase in the relative intensity of the band with a maximum at 800 cm^−1^ compared to the intensity of the band at 890 cm^−1^ in the spectrum of the LAS glass-ceramic suggests that the rate of liquid–liquid phase separation in this glass during this heat treatment is higher than that in the LAS_ox_ glass, i.e., neutral conditions of glass melting speed up the development of liquid–liquid phase separation in this glass. Crystallization of the titanate phases of anatase and tieilite are sped up in the LAS glass ceramized by the heat treatment at the second stage at 780 °C as compared with the LAS_ox_ glass, see Figure 10b. The same tendency remains true for glass-ceramics obtained by heat treatment with the second stage at 900 °C, see Figure 10c. The higher intensity of the anatase peaks in the Raman spectrum of the LAS_ox_ glass-ceramic obtained by heat treatment at 950 °C, see Figure 10d, means that anatase crystallization in the LAS_ox_ glass reaches its maximum and will decrease at higher temperatures while the maximum anatase content in the LAS glass-ceramics was reached at a previous holding temperature. The traces of anatase crystals remain in the LAS_ox_ glass-ceramic obtained by heat treatment at 1000 °C while the LAS glass-ceramic does not show spectral features of this phase, and intensities of peaks assigned to tieilite crystals are higher for the LAS glass-ceramic than for the LAS_ox_ one, see Figure 10e. Figure 10f shows that the LAS glass-ceramic prepared by heat treatment at 1200 °C demonstrates rutile bands of higher intensities as compared with the LAS_ox_ glass-ceramic.

Though Raman spectra of precursor glasses and glasses heat-treated at 680 °C for 6 h are similar to each other, a comparison of the Raman spectra of the materials obtained by two-stage heat treatments unambiguously indicates that, despite the fact that the sequence of transformations in the titania-containing phase is independent of the redox conditions of glass melting, the rate of these transformations is significantly higher at ceramming of the LAS glass.

### 3.4. Morphology Characterization by SEM

**Figure 11:**
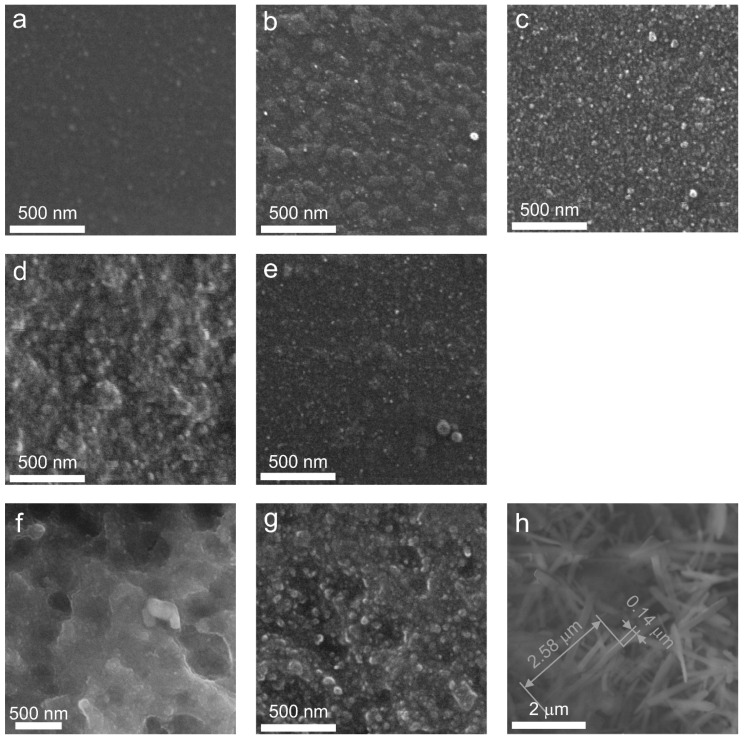
SEM images of the samples of: (**a**) the initial LAS glass; (**b**) the LAS glass after heat treatment at 680 °C; (**c**) the LAS glass ceramized at 680 °C and at 750 °C; (**d**) the LAS glass ceramized at 680 °C and at 780 °C; (**e**) the LAS_ox_ glass ceramized at 680 °C and at 780 °C; (**f**) the LAS glass ceramized at 680 °C and at 800 °C; (**g**) the LAS glass ceramized at 680 °C and at 1000 °C; (**h**) the LAS glass ceramized at 680 °C and at 1200 °C. The holding time is 6 h.

**Figure 12:**
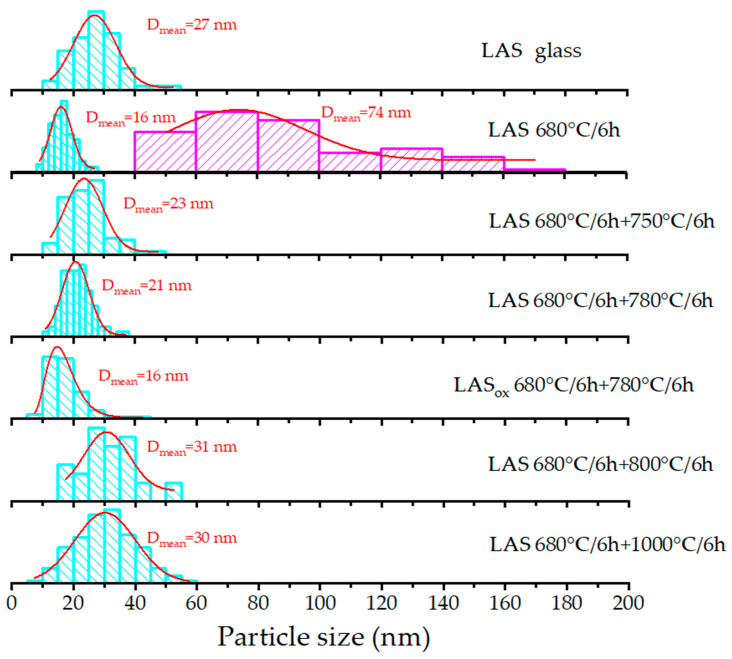
Particle size distributions in the initial LAS glass and LAS and LAS_ox_ glass-ceramics calculated from SEM data presented in Figure 11. Heat-treatment schedules and mean crystallite sizes are listed in figure.

SEM analysis (Figure 11a) reveals the presence of inhomogeneous regions within the bulk of the initial amorphous LAS glass. The calculated size distribution shows a broad profile, with the mean size of the inhomogeneous regions being approximately 27 nm (Figure 12). These inhomogeneities may indicate liquid–liquid phase separation during glass formation. The broad size distribution may arise from overlapping size distributions of chemically distinct regions. Such regions are likely precursors to the crystallization of various phases upon subsequent heat treatments.

Following the single-step heat treatment at 680 °C for 6 h, the LAS glass remains X-ray amorphous. However, its SEM image (Figure 11b) shows increased inhomogeneity, clearly distinguishing two types of regions. The first type consists of small, spherical, bright regions with a narrow size distribution averaging 16 nm (Figure 12). The second predomonant type features larger, irregular, dark regions with an average size of 74 nm (Figure 12).

After two-stage heat treatment at 680 °C for 6 h and 750 °C for 6 h, the large inhomogeneous regions are not seen on the SEM image anymore. Numerous spherical particles appear in the SEM image of the LAS glass-ceramic, averaging 23 nm in size (Figure 11c). The broad shape of the size distribution suggests overlap from multiple chemically distinct particle populations, see Figure 12. XRD analysis confirms the crystallization of the spinel phase with a mean crystallite size of 4.5 nm.

The heat treatment at 680 °C for 6 h and 780 °C for 6 h promotes significant crystallization of the LAS glass. The SEM image of this glass-ceramic is shown in Figure 11d. XRD analysis confirms the formation of three crystalline phases, β-quartz ss, spinel, and Al_2_TiO_5_, see Figure 7c, with mean crystallite sizes of 8 nm, 26 nm and ~6 nm, respectively, see Table 2 and Table 3. The particle size distribution suggests the possible overlap of multiple size distributions corresponding to different phases, see Figure 12. The mean particle size is approximately 21 nm, which is close to the mean size of the predominant crystalline phase of β-quartz ss.

The two-step heat treatment at 680 °C and 800 °C for 6 h results in more extensive crystallization of the LAS glass, see Figure 7e. XRD analysis confirms the presence of crystalline phases of β-quartz ss, spinel, and Al₂TiO₅. The SEM image reveals significant etching of the material, characterized by numerous large caverns, which may indicate silica depletion, see Figure 11f. The mean particle size is 31 nm, see Figure 12, which is also close to the mean size of the predominant crystalline phase of β-quartz ss, which is 25 nm, see Table 2.

Following the two-step heat treatment at 680 °C and 1000 °C for 6 h, XRD analysis shows the formation of β-quartz ss, mullite, and Al_2_TiO_5_. Signs of the residual glass phase become less prominent. The particle size distribution, similar to previous samples, exhibits a broad profile, likely due to overlapping distributions from different phases, with an average particle size of 30 nm. The size of the predominant crystalline phase of β-quartz ss is 28 nm, see Table 2.

Finally, after two-step heat treatment at 680 °C and 1200 °C for 6 h, the SEM image of the opaque LAS sample shows the presence of micron-sized, needle-like crystals and agglomerates of spherical crystals, see Figure 11h. The needle-like crystals are very similar to crystals of β-spodumene ss that were crystallized in the lithium aluminosilicate glass of a different composition during its heat treatment at 1350 °C [12]. XRD analysis confirms the crystallization of β-spodumene (ss), mullite, and Al_2_TiO_5_ phases, see Figure 7e.

The influence of redox conditions of glass melting on the phase assamblage of glass-ceramics is revealed by the comparison of SEM images of LAS and LAS_ox_ glass-ceramics obtained by two-stage heat treatment at 680 °C and 780 °C for 6 h. In accordance with XRD data, see Figure 8a, the SEM image of the LAS_ox_ glass-ceramic shows a significantly reduced crystallinity fraction, see Figure 11e. The particle size distribution for this sample also indicates overlapping distributions, with a mean particle size of 16 nm, which is smaller than the mean particle size of the LAS glass-ceramic. Considering that the main contribution to the increase in mean crystallite size comes from larger crystals of β-quartz ss, the smaller mean crystallite size in the LAS_ox_ glass-ceramic indicates a smaller fraction of these crystals.

### 3.5. Optical Spectroscopy

Light losses in phase separated glasses and glass-ceramics are determined by absorption due to coloring ions and light scattering on the interfaces of regions of inhomogeneity inherent to such materials. It is generally accepted that the coloration of glass-ceramics containing titania as a nucleating agent is mainly caused by intervalent charge transfer transitions between titanium and iron impurity ions in different oxidation states [8,53]. Though the influence of absorption due to the Fe^3+^-Ti^3+^ and Fe^2+^-Ti^4+^ intervalence charge transitions on absorption spectra of our materials cannot be excluded, only absorption due to titanium ions was considered in this study. A similar approach was suggested in [53]. Titanium ions in glasses exist in two oxidation states. The presence of Ti^3+^ and Ti^4+^ ions, and Ti^3+^-Ti^4+^ pairs defines the absorption of glasses under study. Ti^4+^ ion has an electronic configuration of 3d^0^ and does not demonstrate absorption bands due to d–d transitions in the ligand field. Ti^4+^ ions participate in O-Ti^4+^ charge transfer bands positioning in the UV range of spectrum at ~300 nm [54] and in intervalence charge transfer Ti^4+^-Ti^3+^ transitions responsible for coloration in the visible spectral range [55]. Ti^3+^ ions have an electronic configuration of 3d^1^ and demonstrate one broad band in the visible range of spectrum due to the ^2^T_2g_ → E_g_ transition of the Ti^3+^ ions in octahedral symmetry. The band often has a shoulder at the long-wavelength side of the absorption band caused by the Jahn–Teller effect [23,25,26,27,55]. The absorption band of Ti^3+^ ion in tetrahedral (T_d_) symmetry caused by the ^2^T_2g_ → E_g_ transition is located in the near-infrared region of the spectra [55]. The O-Ti^3+^ charge transfer band is expected in UV range of spectrum at ~240 nm [54].

**Figure 13:**
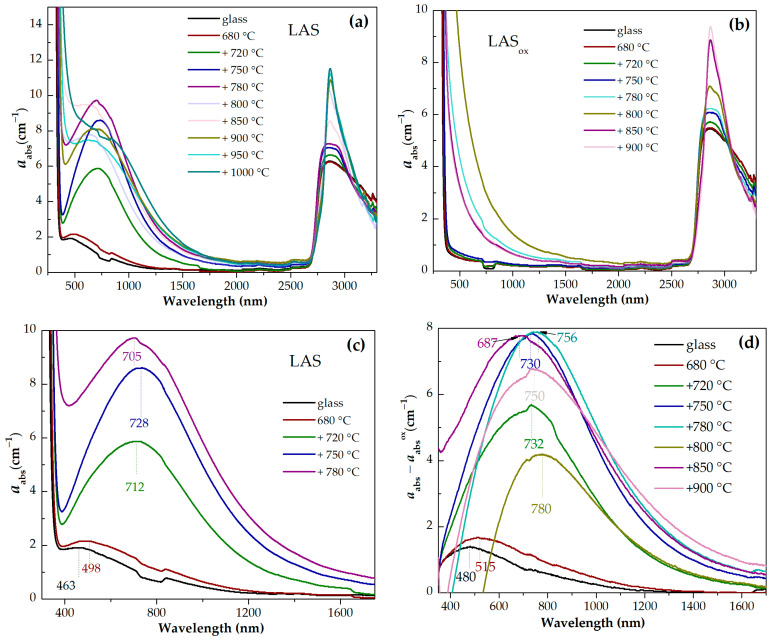

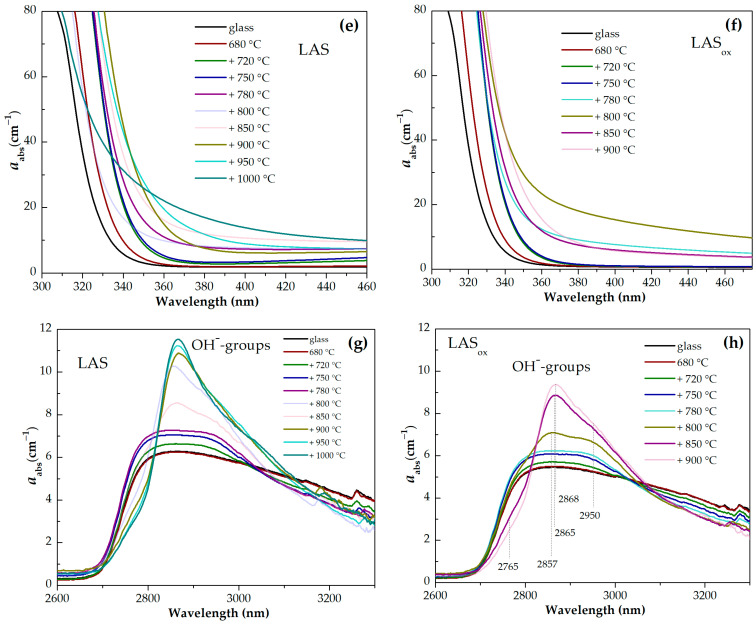
Absorption spectra in different spectral ranges of the samples of: (**a**) the initial LAS glass and glass heat-treated up to 1000 °C in the spectral range from 250 nm to 3300 nm; (**b**) the initial LAS_ox_ glass and glass heat-treated up to 900 °C in the spectral range from 250 nm to 3300 nm; (**c**) the LAS glass and glass heat-treated up to 780 °C in the spectral range from 300 nm to 1750 nm; (**d**) the subtracted spectra; (**e**) the LAS glass and glass heat-treated up to 1000 °C in the spectral range from 300 nm to 460 nm; (**f**) the LAS_ox_ glass and glass heat-treated up to 900 °C in the spectral range from 300 nm to 475 nm; (**g**) the LAS glass and glass heat-treated up to 1000 °C in the spectral range from 2600 nm to 3300 nm; (**h**) the LAS_ox_ glass and glass heat-treated up to 900 °C in the spectral range from 2600 nm to 3300 nm. The duration of each hold is 6 h.

**Figure 14:**
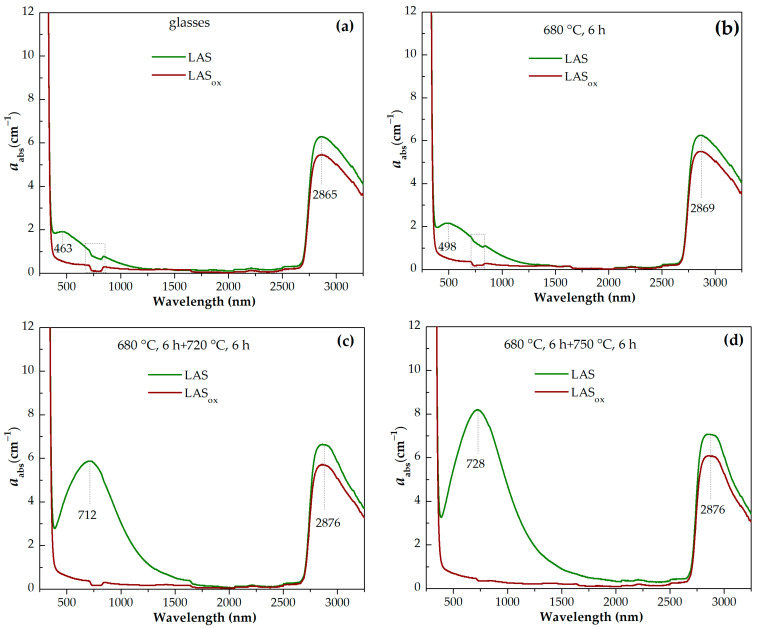

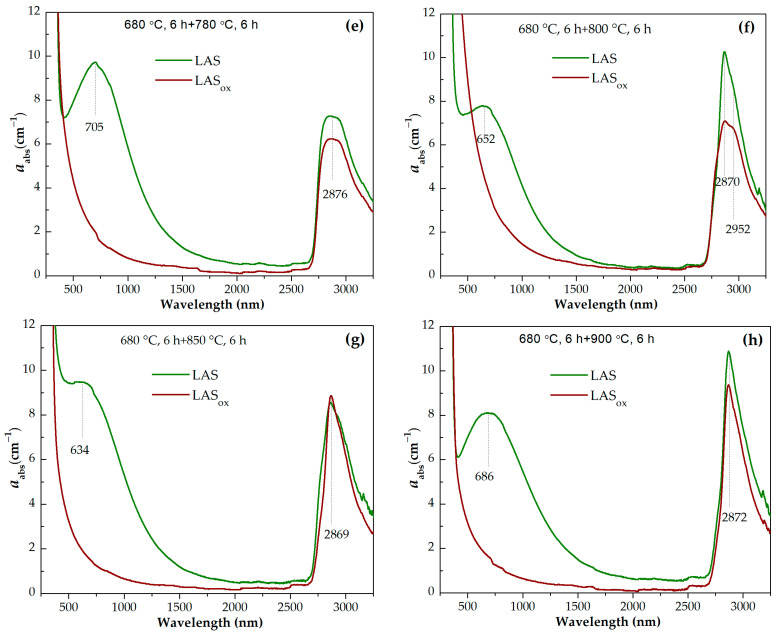
Absorption spectra of the samples of: (**a**) the initial LAS and LAS_ox_ glasses; (**b**) the LAS and LAS_ox_ glasses heat-treated at 680 °C; (**c**) the LAS and LAS_ox_ glasses heat-treated at 680 °C + 720 °C; (**d**) the LAS and LAS_ox_ glasses heat-treated at 680 °C + 750 °C; (**e**) the LAS and LAS_ox_ glasses heat-treated at 680 °C + 780 °C; (**f**) the LAS and LAS_ox_ glasses heat-treated at 680 °C + 800 °C; (**g**) the LAS and LAS_ox_ glasses heat-treated at 680 °C + 850 °C; (**h**) the LAS and LAS_ox_ glasses heat-treated at 680 °C + 900 °C. The duration of each hold is 6 h.

Absorption spectra of initial and heat-treated glasses are shown in Figure 13a–h. For the convenience of comparison, the spectra of the LAS and LAS_ox_ glasses and glass-ceramics obtained by the same heat-treatment schedule are presented in Figure 14a–h. The spectra are formed by the absorption edge in the UV spectral range, see Figure 13e,f, intense absorption of OH^−^ groups spanning from 2700 nm to 3300 nm, see Figure 13g,h, and light losses in the visible and near-IR spectral range, see Figure 13a–c, which have different origin in the LAS and the LAS_ox_ glass-ceramics. Note that the LAS_ox_ glass-ceramics obtained by heat treatments at 950 °C and 1000 °C at the second stage cracked during heat treatment, and their spectra were not recorded.

For the initial LAS glass, the UV absorption edge is observed at ~330 nm. In the spectrum of the glass heat-treated at the nucleation stage at 680 °C for 6 h it moves to longer wavelengths by 5 nm. In the spectrum of glass-ceramics obtained by the two-stage heat treatment with a temperature of 720 °C at the second stage, the position of the absorption edge is found at ~343 nm and it is nearly the same after heat treatments at 750 °C and 780 °C. After heat treatment with a temperature of 800 °C at the second stage, the absorption edge shifts to shorter wavelengths, to 334 nm. After increasing the temperature of the second stage to 850 °C and 900 °C, the absorption edge shifts again to longer wavelengths. Its position is equal to 347 nm and 351 nm, respectively. After heat treatments at 950 °C and 1000 °C, the position of the absorption edge shifts again to shorter wavelengths, see Figure 13e.

The position of the absorption edge for the LAS_ox_ glass and its variation with the heat-treatment temperature are surprisingly similar to those of the LAS glass, see Figure 13e,f and Figure 14a–e,g,h. The position of the absorption edge differs only for glass-ceramics obtained by the heat treatment at the second stage at 800 °C, see Figure 14f. The absorption edge in the spectrum of this LAS_ox_ glass-ceramic is shifted to longer wavelengths compared to its position in the spectrum of the LAS sample. The nonmonotonic transmittance variation during the crystallization of glasses inclined to liquid–liquid phase separation was described in ref. [56] and assigned to incoherent scattering that takes place in a material containing amorphous and crystallized regions of inhomogeneity. The authors of ref. [56] demonstrated that the extinction coefficient can reach a maximum when the crystallinity fraction is 0.5–1.0 and then decrease due to the presence of elements of ordering in the relative position of the crystallites.

The same raw materials were used for the preparation of both glasses, which means that the iron content in both glasses was similar. The absorption edge in precursor glass and in glass-ceramics originates from O^2−^→Ti^4+^ and O^2−^→Ti^3+^ charge transfer bands [54]. Taking into account that the O^2−^→Ti^4+^ charge transfer band is located at longer wavelengths than the O^2−^→Ti^3+^ one, it can be suggested that the content of Ti^4+^ ions in the LAS and the LAS_ox_ glasses is very similar, i.e., the content of Ti^3+^ in the LAS glass is rather low. In glass-ceramics containing β-quarts ss, scattering losses are superimposed with the absorption edge.

A broad absorption band is found in the spectrum of the LAS precursor glass. Its intensity increases after heat treatment at the nucleation stage and with the increasing of the heat-treatment temperature at the second stage up to 780 °C, see Figure 13a,c. After further heat treatments the absorption band becomes broader, and its intensity somewhat decreases. The appearance of this absorption band can be explained by Ti^3+^ ions distributed between different amorphous and crystalline phases. Since the absorption edge and light scattering in the obtained multiphase materials are superimposed on the short-wave part of the absorption spectrum, absorption due to these losses was subtracted from the experimental absorption spectra of the LAS samples using the corresponding absorption spectra of LAS_ox_ samples for substraction. The difference spectra are shown in Figure 13d. The spectrum of the precursor glass is a typical absorption spectrum of Ti^3+^ ions in silicate glasses with a broad peak with a maximum at ~480 nm attributed to d–d transition ^2^T_2g_→^2^E_g_ of 3d^1^ electron of Ti^3+^ ions in octahedral coordination in the ligand field affected by the Jahn–Teller effect [25,26,27]. The tail with a maximum at ~800 nm is assigned to intervalence charge transfer in Ti^3+^-Ti^4+^ pairs [57,58]. The absorption intensity increases after the preliminary heat treatment, the band maximum shifts to ~500 nm, which is connected with the participation of Ti^3+^ ions in the liquid–liquid phase separation and entering into the aluminotitanate amorphous regions. With the increase in the heat-treatment temperature at the second stage, the broadband absorption in the visible and near-IR region intensifies, and its maximum shifts to ~730 nm in the spectra of glass-ceramics obtained by heat treatments at 750 °C and 780 °C at the second stage. Similar to the absorption of Ti^3+^ ions in corrund crystals [59], this absorption is attributed to Ti^3+^ ions in the ligand field of octahedral symmetry (the shoulder at ~480 nm) and Ti^3+^-Ti^4+^ pairs in crystals of γ-Al_2_O_3_. In the spectrum of corrundum, the Ti^4+^ ions in Al^3+^ sites are considered to be charge-compensated by Al vacancies with one vacancy for every three Ti^4+^ ions [60]. An increase of the lattice parameters of γ-Al_2_O_3_ with heat-treatment temperature found by the XRD analysis can be due to the entering of the crystals of γ-Al_2_O_3_ by Ti^3+^ ions (ionic radius in octahedral coordination is 0.81 Å) and Ti^4+^ ions (ionic radius in octahedral coordination is 0.745 Å). Note that the ionic radii of Al^3+^ in octahedral coordination is 0.675 Å.

The absorption band shape changes with the raising of the heat-treatment temperature. The spectrum is still mainly formed by the absorption of Ti^3+^-Ti^4+^ pairs. Taking into account the crystallization of β-quartz ss and tieilite, Al_2_TiO_5_, this change in the shape of the absorption band can be connected with the distribution of titanium ions between the crystalline phases. Spinel does not crystallize in glass-ceramics obtained by heat treatments at 850–1000 °C at the second stage. So the broadband spectra of glass-ceramics obtained by these heat treatments are connected with titanium ions in β-quartz ss and tieilite.

The light losses in the LAS_ox_ glass-ceramics are mostly caused by light scattering, which has a non-monotoneous dependence on the heat-treatment temperature, as was mentioned above.

Figure 13g,h show wide asymmetric absorption bands in the spectral region from ~2700 nm to 3300 nm caused by the presence of OH-groups in glasses and glass-ceramics. Heat treatment at the nucleation stage at 680 °C for 6 h has no effect on the shape and intensity of this band. Two-stage heat treatments with a temperature from 720 °C to 780 °C at the second stage cause a successive change in the spectrum of OH-groups. The intensity in the range from 2750 nm to 3050 nm increases and the intensity in the range from 3050 nm to 3300 nm decreases. Further narrowing of the OH-groups absorption band and growth of its intensity is observed in spectra of glass-ceramics obtained during heat treatments in the range of crystallization temperatures from 800 °C to 1000 °C. Comparison of the absorption bands of OH-groups in glasses melted in different redox conditions and in corresponding glass-ceramics showed that the intensities of the absorption bands of OH-groups are higher in inital and heat-treated LAS glass than in LAS_ox_ samples. The absorption band shape is the same for the materials obtained by the same heat-treatment shedule, see Figure 14a–h, which is explained by the similarity of their phase compositions.

### 3.6. Estimation of the Coefficient of Thermal Expansion

Table 4 lists the coefficients of thermal expansion (CTE) for the initial and heat-treated glasses of the LAS and the LAS_ox_ compositions in the temperature range between 20 °C and 320 °C. The CTE values of precursor glasses are similar and equal to ~4.3 × 10^−6^ K^−1^. After the nucleation heat treatment at 680 °C for 6 h the CTE values slightly decrease to become ~4.1 × 10^−6^ K^−1^. There is a gradual increase of CTE values with the increase in the ceramming temperature to 720 °C and then to 750 °C. Glass-ceramics based on spinel nanocrystals and prepared by the consequent three-stage heat treatment schedule 680 °C, 6 h + 720 °C, 6 h + 750 °C, 6 h have the highest CTE value of ~5.2 × 10^−6^ K^−1^. The CTE value starts to decrease as the first portions of β-quartz ss crystallize (heat treatment with the last hold at 780 °C for 6 h) and reaches the lowest value of ~0.3 × 10^−6^ K^−1^ for the glass-ceramics obtained by ceramming at the last stage of heat treatment at 950 °C for 6 h. After heat treatment of the precursor glass at 1200 °C, the CTE increases again to a value of 1.46 × 10^−6^ K^−1^ due to the crystallization of β-spodumene ss. The dependences of the change in the CTE value on the temperature of heat treatment for samples obtained by the crystallization of glasses melted under neutral and oxidizing conditions are similar, which can be explained by the similarity of their phase assemblage.

## 4. Discussion

Glasses melted in neutral and oxidizing conditions are X-ray amorphous and inhomogeneous according to SEM microscopy findings. Inhomogeneity regions have a broad size distribution with a mean size of 27 nm. In accordance with previous studies [19,20], it is suggested that liquid–liquid phase separation occurs during glass melt casting and annealing. Absorption spectra of glasses melted in different redox conditions are different by the appearance of a broadband absorption in the LAS glass mainly assigned to Ti^3+^ ions in distorted octahedral coordination and Ti^3+^-Ti^4+^ pairs in the glass structure. One may suggest that these ions are distributed between the lithium aluminosilicate glass matrix and amorphous regions of inhomogeneity composed of octahedrally coordinated titanium and aluminum ions even with the formation of Ti^3+^-Ti^4+^ clusters [18]. Based on the similarity of XRD patterns, Raman spectra and glass transition temperatures, it was speculated that glasses melted in neutral and oxidizing conditions have a similar structure because of the low content of Ti^3+^ ions. Nevertheless, the role of Ti^3+^ ions in liquid–liquid phase separation and crystallization of LAS glass is clearly seen.

The glasses obtained by heat treatment at 680 °C for 6 h do not contain any crystalline phase, their XRD patterns are similar, and their Raman spectra are near similar to each other and to those of precursor glasses. However, there is a tremendous difference in the behavior of their DSC curves, their morphology revealed in the SEM study and their absorption spectra as compared with those of precursor glasses. It allows us to conclude that liquid–liquid phase separation continues to develop in both glasses during the nucleation heat treatment.

According to data of SEM analysis, two types of amorphous regions with mean sizes of 16 and 74 nm are formed in the glass during heat treatment at 680 °C for 6 h. A similar structure formation was not found in previously studied glasses of the lithium aluminosilicate system. The formation of large amorphous aluminotitanate regions of inhomogeneity in magnesium ([20] and refs therein) and zinc ([20] and refs therein) aluminosilicate glasses nucleated by titania was previously revealed by a combination of small-angle X-ray scattering and Raman spectroscopy data. Those materials also contained smaller size phase-separated regions enriched in aluminates of corresponding cations ([20] and refs therein). One can suggest a similar character of phase separation in glasses under study and suppose that Ti^3+^ ions are distributed between large aluminotitanate amorphous regions, smaller size aluminate regions and residual glass.

Judging by the data of Raman spectroscopy, glasses cerammed using a two-stage heat treatment with a temperature at the second hold of 720 °C have a well-developed liquid–liquid phase separated structure. During this phase separation, spinel nanocrystals appear in both glasses, and the composition of the residual lithium aluminosilicate glass slightly changes, which is manifested by a change in the position of the amorphous halo in the XRD pattern and the position of the band at ~480 cm^−1^ in the Raman spectrum. Spinel is the sole crystalline phase in transparent glass-ceramics obtained by heat treatments at the second stage at 720 °C and 750 °C. Upon crystallization of spinel, titanium ions Ti^3+^ enter its structure, which is clearly seen in the absorption spectra of the LAS glass-ceramics. The change of the position of the maximum of absorption band to longer wavelengths suggests that absorption spectrum in glass-ceramics is mainly formed by the Ti^3+^-Ti^4+^ intervalent charge transfer band. This is a spectroscopic confirmation of the presence of Ti^4+^ ions in the spinel structure.

Therefore, based on the DSC, SEM and XRD data, it was concluded that preliminary heat treatment at 680 °C, resulting in the formation of a reach phase-separated structure, provokes spinel crystallization during further heat treatments between 720 °C and 800 °C. According to the similarity of absorption spectra of these glass-ceramics and spectra of Ti^3+^-doped Al_2_O_3_, it was speculated that Ti^3+^ ions enter into spinel nanocrystals. Taking into consideration that, according to the findings of the Raman spectroscopy, the rate of phase transformations in the titania-containing phase in glass melted in neutral conditions is significantly higher than in glass melted in oxidizing conditions, it was suggested that Ti^3+^ ions become a component of amorphous aluminotitanate phase-separated regions.

Previously, the decrease in intensity of the first broad peak in the DSC curves of glasses nucleated at 700 °C and its near disappearance from DCS curves of glasses preliminarily heat-treated at 720 °C for 6 h was mentioned. The sample preliminarily heat-treated at 720 °C already contained spinel nanocrystals. Therefore, in this sample only high temperature phases of β-quartz ss, tieilite and β-spodumene ss crystallized, which resulted in the appearance of the second and the third peaks and the absence of the first peak on the DSC curve.

A specific feature of the morphology of the materials under study is the similarity of the mean size of inhomogeneous regions in the precursor glass and in glass-ceramics obtained by two-stage heat treatments at temperatures up to 1000 °C. Sizes of β-quartz ss crystallized in both glasses in the temperature range from 780 °C to 1000 °C are similar to each other. Our findings are in line with the results presented in ref. [38] and devoted to the study of the lithium aluminosilicate glass nucleated by a mixture of TiO_2_ and ZrO_2_, where the constancy of the size of β-quartz ss crystallized in the temperature range from 860 °C to 960 °C was revealed and discussed.

The comparison of XRD patterns of the LAS and the LAS_ox_ glass-ceramics obtained at 1200 °C demonstrates that LAS glass-ceramic contains a larger fraction of rutile and a smaller fraction of mullite than the LAS_ox_ glass-ceramic. Previously it was revealed the difference in lattice parameters of rutile crystallized in zinc aluminosilicate glasses nucleated by TiO_2_ and melted in different redox conditions [34]. In ref. [34] this difference was explained by the presence of Ti^3+^ ions in the rutile structure. Here, it is suggested that in lithium aluminosilicate glass-ceramics Ti^3+^ ions also participate in the formation of rutile nanocrystals, which results in the facilitation of their crystallization. Note that the role of small additives of transition metal ions in kinetics of crystallization in glass-ceramics was discussed in ref. [18].

The rate of phase transformations resulted in the crystallization of β-quartz ss is significantly higher during the heat treatment of the glass melted in neutral conditions. However, the sequence of phase transformations and phase assemblage of glass-ceramics melted in different redox conditions are independent of the redox conditions of glass melting. This is very promising for the development of transparent lithium aluminosilicate glass-ceramics with close-to-zero thermal expansion coefficient and doped with various functional ions in the lower oxidation states. Crystallization of β-quartz ss leads to a decreasing of the value of the thermal expansion coefficient. The lowest coefficient of thermal expansion was obtained by the heat treatment at 950 °C. It was ~0.3 × 10^−6^ K^−1^.

## 5. Conclusions

The glass of the lithium aluminosilicate system containing titanium oxide as a nucleating agent was melted with and without the addition of As_2_O_3_, i.e., under oxidizing and neutral conditions. The glasses were heat-treated in the temperature range from 680 °C to 1300 °C to obtain glass-ceramics. Glass-ceramics developed from glasses melted without the addition of As_2_O_3_ are black-colored, and those fabricated from glasses melted with addition of As_2_O_3_ are colorless.

Transparent glass-ceramics based on β-quartz ss and/or γ-Al_2_O_3_ nanocrystals and opaque glass-ceramics based on β-spodumene ss nanocrystals were obtained. Crystallization of β-quartz ss was accompanied by nanocrystals of tieilite, Al_2_TiO_5_. Crystallization of β-spodumene ss was accompanied by tieilite, mullite (at 1100 °C) and rutile (at 1200 °C).

Heat treatment at the nucleation stage causes crystallization of γ-Al_2_O_3_ with a spinel structure and an increase in the crystallization temperature of β-quartz ss and Al_2_TiO_5_ and decrease in the temperature of β-spodumene ss crystallization. Glasses melted under neutral conditions lose transparency at higher secondary heat-treatment temperatures than glasses melted under oxidizing conditions.

X-ray amorphous precursor glasses have an inhomogeneous structure with a mean size of regions of inhomogeneity of ca. 27 nm. Preliminary heat treatment at 680 °C results in the formation of a reach bimodal liquid–liquid phase separated structure with mean sizes of inhomogeneous regions of ca. 16 nm and 74 nm. Titanium ions enter both types of inhomogeneous regions. An appearance of a bimodal structure of inhomogeneous regions in the result of heat treatment at the nucleation stage suggests the development of a three-phase immiscibility in the lithium aluminosilicate glass with a high excess of alumina over lithium oxide, i.e., the formation of two amorphous phases, the aluminotitanate phase and the aluminate phase, dispersed in a high-silica matrix. It is the first time that three-phase immiscibility is revealed in glasses of the lithium aluminosilicate system.

It is the first time that the formation of nanocrystals of γ-Al_2_O_3_ with a spinel structure appeared during heat treatments at the second stage in the temperature interval between 720 °C and 800 °C was documented. Titanium ions are located in the spinel structure as Ti^3+^ ions in the ligand field of octahedral symmetry and in Ti^3+^-Ti^4+^ pairs. Ti^3+^ ions also enter amorphous aluminotitanate phase-separated regions and facilitate phase transformations in this phase with formation of tieilite, anatase and rutile.

The phase assemblage and sequence of phase transformations is independent of the redox conditions of glass melting; however, the rate of these transformations is significantly higher when ceramming the glass melted in neutral conditions. This is very promising for the development of transparent glass-ceramics with close-to-zero thermal expansion coefficient and doped with various functional ions in lower oxidation states.

The variation of the coefficient of thermal expansion with heat-treatment temperature reflects the variation of the phase composition of the developed materials, does not depend on the redox conditions of glass melting and reaches as low value as ~0.3 × 10^−6^ K^−1^ for glass-ceramics based on nanocrystals of β-quartz ss.

## Figures and Tables

**Table 1:** Characteristic temperatures of initial quenched and heat-treated glasses shown in DSC curves.

Heat-Treatment Regime	T_g_, °C	T_on1_, °C	T_max1_, °C	T_on2_, °C	T_max2_, °C	T_max3_, °C
**The LAS glass**						
quenched	698	-	-	847	867	1175
680 °C, 6 h	699	810	839	882	909	1142
700 °C, 6 h	705	764	807	878	900	1135
720 °C, 6 h	708	765	815	871	890	1139
**The LAS_ox_ glass**						
quenched	699	-	802	851	874	1186
680 °C, 6 h	695	799	831	876	901	1136
700 °C, 6 h	704	779	809	879	899	1141
720 °C, 6 h	708	755	828	876	900	1136

**Table 2:** Lattice parameters and mean crystallite sizes of γ-Al_2_O_3_, β-quartz ss and β-spodumene ss in LAS and LAS_ox_ glass-ceramics.

Heat-Treatment Regime	γ-Al_2_O_3_		β-Quartz ss			β-Spodumene ss		
	*a*, Å	D, nm	*a*, Å	*c*, Å	D, nm	*a*, Å	*c*, Å	D, nm
**The LAS glass**								
680 °C, 6 h + 720° C, 6 h								
680 °C, 6 h + 750 °C, 6 h	7.918	4.5						
680 °C, 6 h + 780° C, 6 h	7.921	8.0	5.207	5.336	26			
680 °C, 6 h + 800 °C, 6 h	7.925	14.0	5.196	5.350	25			
680 °C, 6 h + 850 °C, 6 h			5.189	5.370	22			
680 °C, 6 h + 1000 °C, 6 h			5.186	5.362	28			
680 °C, 6 h + 1100 °C, 6 h						7.552	9.145	45
680 °C, 6 h + 1200 °C, 6 h						7.541	9.143	45
**The LAS_ox_ glass**								
680 °C, 6 h + 720° C, 6 h								
680 °C, 6 h + 750 °C, 6 h	7.916	7.3			28			
680 °C, 6 h + 780° C, 6 h	7.915	9.4	5.207	5.319	26			
680 °C, 6 h + 800 °C, 6 h	7.921	10.3	5.197	5.337	26			
680 °C, 6 h + 850 °C, 6 h			5.192	5.365	21			
680 °C, 6 h + 1000 °C, 6 h			5.187	5.368	27			
680 °C, 6 h + 1100 °C, 6 h						7.550	9.153	36
680 °C, 6 h + 1200 °C, 6 h						7.551	9.150	36

**Table 3:** Lattice parameters and mean crystallite sizes of Al_2_TiO_5_ in LAS and LAS_ox_ glass-ceramics.

Heat-Treatment Regime	Al_2_TiO_5_									
	The LAS Glass					The LAS_ox_ Glass				
	*a*, Å	*b*, Å	*c*, Å	V, Å^3^	D, nm	*a*, Å	*b*, Å	*c*, Å	V, Å^3^	D, nm
680 °C, 6 h + 780° C, 6 h	3.594	9.356	9.557	321.3	6.2	3.593	9.541	9.577	321.4	8.0
680 °C, 6 h + 800 °C, 6 h	3.595	9.365	9.564	322.0	7.1	3.594	9.358	9.577	322.1	8.7
680 °C, 6 h + 850 °C, 6 h	3.592	9.378	9.560	322.0	8.3	3.595	9.385	9.589	323.5	10.2
680 °C, 6 h + 900 °C, 6 h	3.596	9.373	9.570	322.6	9.0	3.594	9.418	9.619	325.6	12.0
680 °C, 6 h + 950 °C, 6 h	3.597	9.454	9.590	326.1	9.4	3.594	9.421	9.628	326.0	12.0
680 °C, 6 h + 1000 °C, 6 h	3.598	9.456	9.624	327.4	12.2	3.594	9.457	9.633	327.4	13.7
680 °C, 6 h + 1100 °C, 6 h	3.596	9.456	9.663	328.6	18.8	3.595	9.460	9.677	329.1	19.4
680 °C, 6 h + 1200 °C, 6 h	3.591	9.488	9.716	331.0	44.1	3.592	9.489	9.708	330.9	42.0

**Table 4:** Coefficients of thermal expansion (CTE) for precursor and heat-treated glasses of the LAS and the LAS_ox_ compositions in the temperature range from room temperature to 320 °C.

Heat-Treatment Regime	CTE_20-320_ (×10^−6^ K^−1^)	
	LAS	LAS_ox_
Precursor glasses	4.35 ± 0.08	4.25 ± 0.08
680 °C, 6 h	4.19 ± 0.08	4.21 ± 0.08
680 °C, 6 h + 720 °C, 6 h	4.82 ± 0.09	4.57 ± 0.09
680 °C, 6 h + 720 °C, 6 h + 750 °C, 6 h	5.36 ± 0.10	5.18 ± 0.10
680 °C, 6 h + 720 °C, 6 h + 750 °C, 6 h + 780 °C, 6 h	4.30 ± 0.08	4.85 ± 0.09
680 °C, 6 h + 780 °C, 6 h	-	4.85 ± 0.10
720 °C, 6 h + 800 °C, 6 h	1.15 ± 0.02	1.24 ± 0.01
720 °C, 6 h + 850 °C, 6 h	0.61 ± 0.01	0.45 ± 0.08
680 °C, 6 h +720 °C, 6 h +750 °C, 6 h +950 °C, 6 h	0.30 ± 0.01	0.19 ± 0.01
680 °C, 6 h +1200 °C, 6 h	1.49 ± 0.03	1.42 ± 0.03

## Data Availability

The original contributions presented in this study are included in the article. Further inquiries can be directed to the corresponding author.
